# A Review on Low-Cost Microwave Doppler Radar Systems for Structural Health Monitoring

**DOI:** 10.3390/s21082612

**Published:** 2021-04-08

**Authors:** Davi V. Q. Rodrigues, Changzhi Li

**Affiliations:** Department of Electrical and Computer Engineering, Texas Tech University, Lubbock, TX 79409, USA; changzhi.li@ttu.edu

**Keywords:** Doppler radar, displacement measurement, low-cost radar, micro-Doppler, portable radar, remote sensing, radar network

## Abstract

Portable, low-cost, microwave radars have attracted researchers’ attention for being an alternative noncontact solution for structural condition monitoring. In addition, by leveraging their capability of providing the target velocity information, the radar-based remote monitoring of complex rotating structures can also be accomplished. Modern radar systems are compact, able to be easily integrated in sensor networks, and can deliver high accuracy measurements. This paper reviews the recent technical advances in low-cost Doppler radar systems for phase-demodulated displacement measurements and time-Doppler analysis for structural health information, including digital signal processing and emerging applications related to radar sensor networks.

## 1. Introduction

Radars have been employed since 1940s as surveillance systems [1]. In the past, they were mainly used in the military due to high costs and bulky sizes. Thanks to the fast and significant advancements in the semiconductor industry, radars are being miniaturized and assembled on printed circuit boards (PCB) or even integrated into a single chip with antenna-on-chip/antenna-in-package technologies [2,3,4,5]. In the last decades, portable short-range radars have been investigated for human and animal vital signs, remote voice recording, gait analysis, fall detection, gesture characterization, occupancy sensing, and security applications [6,7,8,9,10,11,12,13,14,15,16,17,18,19]. Biomedical Doppler radars have also been employed in cancer radiotherapy for respiratory gating and tumor tracking for motion-adaptive radiotherapy [20,21,22]. In addition, short-range radars can be used to provide time-frequency analysis of microwave signals backscattered by rotating structures such as wind turbines [23,24,25,26,27,28].

Structural health monitoring (SHM) is the process of continuous observation of a structure or mechanical system using one or multiple sensors to provide information about its true capacity, which is altered by age and/or accidental damage. By sensing low-frequency small-amplitude mechanical vibration or deflection of structures, radars may also be successfully employed for the SHM of infrastructures. As the aging of worldwide infrastructures raises concerns and the appearance of new structures forces researchers and engineers to look for alternative solutions for SHM measurements and the improvement of existing ones, private industries and government organizations demand technologies that are able to detect structural damage at the earliest possible time to avoid life-threatening situations and economical losses.

Several technologies targeting SHM have been proposed in the past decades. Accelerometers are commonly used to evaluate the infrastructure health condition and to extract damage-sensitive features because they are relatively cost-effective and can be readily instrumented. Nevertheless, the double integration of the acceleration data makes displacement measurements susceptible to integral drift errors [29]. Strain sensors, laser displacement sensors, and vision-based systems are also representatives of nonintrusive solutions for SHM applications. However, these sensors present practical limitations. The measurements retrieved by strain gauges are sensitive to temperature variations, and they may need periodical calibration. Laser displacement sensors are sensitive to the measurement range and the structure’s surface condition. Vision-based systems demand large data storage, high computational load for image recognition and are not robust against ambient light.

On the other hand, radars can make use of different types of waveforms for the targeted application. Continuous-wave (CW) radars have simple architecture, allowing for easier integration and lower power consumption, which makes them appealing for portable applications. In a basic CW radar system, the radio frequency (RF) wave is radiated, and an echo returns after being backscattered by a surrounding target. If the target is moving, a shift in the received radar signal due to the Doppler effect is observed. The target’s range can only be assessed by modulated CW radars. Modulated CW radars such as frequency-modulated continuous-wave (FMCW) radars and stepped-frequency continuous-wave (SFCW) radars are popular candidates for applications that require range and/or Doppler information. In contrast, unmodulated CW radars, widely known as Doppler radars, do not have range discrimination capability. Nonetheless, Doppler radars can measure time-varying small-amplitude periodical motion with high accuracy [2].

The most common radars employed on structural condition monitoring are the ground-based interferometric (GBI) radars. Their use for SHM applications has a relatively long history. The inspiration for the utilization of GBI radars on the monitoring of structures such as building or bridges came from the success of spaceborne synthetic aperture radar (SAR) radar systems, which operate at high orbits and detect ground changes based on the phase information of radar images [30,31]. During onsite testing, these sensors are typically mounted on a tripod and pointed towards bridges, landslides, towers, and dams [32,33,34,35,36,37,38,39,40,41,42,43,44,45,46,47,48,49,50,51,52,53,54,55]. The main difference between GBIR and other portable radar sensors is their relatively large detection range due to the use of bulky, high directivity antennas and waveguide-based components [38]. By transmitting and receiving electromagnetic waves at microwave frequencies, they can remotely detect small displacements of targets using the interferometric technique, and they are also able to distinguish the real displacement of targets of interest from clutter since the vast majority of GBI radar systems employs stepped-frequency continuous-wave (SFCW) or frequency-modulated continuous-wave (FMCW) radar sensors. These systems can operate without any angular resolution or with angular resolution obtained through the rotation of the radar or the movement of the radar along a linear mechanical guide. GBI radars are powerful tools on the estimation of vibration parameters of structures with large areas (bridges, mines, buildings). Bridge monitoring using portable GBI radars dates back to the 2000s [32]. The evaluation of the bridge displacement along the radial direction by an SFCW or FMCW radar systems starts by choosing the range bin associated with different parts of the structure. After the selection of the range bin, the displacement of the target is recovered by demodulating the phase variations of the received signal during the detection time. Another important parameter of modulated wave radar is the range resolution, which is a function of the transmitted bandwidth and is the minimum distance to resolve two or more adjacent targets on the same bearing.

Monostatic radars can only detect motion along the radial direction, and the movement of real large targets such as buildings or bridges consists of more than one component. To address the challenge of simultaneously measuring displacements along different directions, the most recent work on SHM based on GBI radars proposed a multi-monostatic 17.2-GHz FMCW radar for the remote monitoring of bridges [55]. They employed two different interferometric radars placed at different positions to measure two components of a bridge’s deck motion. The used radar system was a modified version of a multiple-input multiple-output (MIMO) GBI radar (IBIS-FM MIMO by IDS Company) that operated with two pairs of transmitting (Tx) and receiving (Rx) antennas (four possible baseband channels). Only two channels were effectively used to retrieve the displacements at two different positions (23 m and 33 m away from the main radar) on the bridge. RF cables were utilized to connect the second pair of Tx/Rx antennas to the main radar system, allowing for the multi-monostatic radar architecture. The radar operated sequentially in a single channel modality, but the time duration between four acquisitions was relatively short (5–12 ms), especially for SHM applications. The cable loss and the time shifts were compensated by low noise amplifiers and by digital signal processing techniques, respectively. The authors chose the 127-m long Varlungo Bridge in Firenze, Italy, to conduct the full-scale experiments. The vehicular traffic provoked the bridge vibration, but a significant change on the displacement was only observed when a truck moved on the bridge. With this strategy, the authors were able to retrieve the displacement vector (*y*-*z* plane) and the natural frequencies for the two different radar targets. However, no discussion was made towards modal analysis measurements. In addition, the proposed method relies on choosing an appropriated distance between the main radar and the second pair of Tx/Rx antennas, which must be large enough to allow the evaluation of the vector displacement using the two motion components. The radar had achieved submillimeter accuracy during measurements in a controlled environment with an oscillating corner reflector placed 12.88 m away from the main radar and 7.33 m away from the second pair of antennas. Seismic accelerometer measurements (PCB 393B31 by PCB piezotronics) provided the ground truth.

This review mainly focuses on recent advancements on portable, board-level Doppler radar for structural health monitoring. Interested readers on GBI radar-based SHM are encouraged to refer to papers cited in this section for more information on ground-based radar technologies and applications [32,33,34,35,36,37,38,39,40,41,42,43,44,45,46,47,48,49,50,51,52,53,54,55]. The remainder of this paper is organized as follows. Section 2 addresses the theoretical formulation of low-cost Doppler radar, including the arctangent demodulation, the time-frequency analysis technique, and data processing techniques used for field SHM. The recent advancements on vibration based SHM using low-cost Doppler radar are described in Section 3. In Section 4, SHM based on time-frequency analysis is reviewed. Finally, the outlook for portable microwave radar sensing for SHM is discussed in Section 5.

## 2. Theory of Low-Cost Doppler Radars for Structural Health Monitoring

Doppler radars emit a single-tone microwave signal of frequency $f_{t}$. The reflected radio-frequency signal is frequency/phase-modulated due to the Doppler effect assuming that the target is moving. The microwave frequency associated with the translational speed v of a point-scatterer target is calculated as $f_{r}=f_{t}\left(1+v/c\right)/\left(1-v/c\right)$, and can be easily retrieved by spectral analysis. If the target consists of several moving parts, the contributions of various point-scatterers produce micro-Doppler signatures, which can be exploited for the extraction of other parameters not related to the main target’s movement [23]. For example, when one does hand gestures in front of a radar sensor, not only will the Doppler frequencies associated with the hand’s movement be captured, but also the frequencies associated with the movements of other parts of the human body such as the arm and the elbow [17,18].

**Figure 1:**
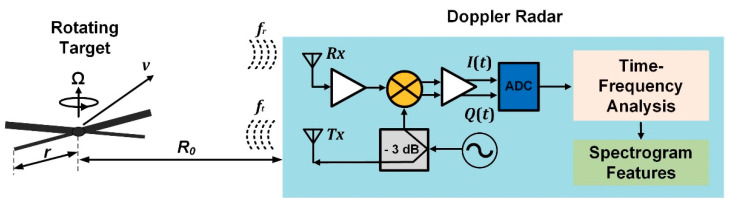
Block diagram for structural health monitoring (SHM) based on low-cost Doppler radars using time-frequency analysis of micro-Doppler signatures.

Assuming that a simple homodyne Doppler radar illuminates a structure comprised of rotating blades, as seen is Figure 1, the reflected signal is phase-modulated by their movement. The returned echoes are mixed with the same transmitted signal by a quadrature-mixer to generate in-phase (*I*(*t*)) and quadrature-phase (*Q*(*t*)) baseband responses. The analytic form of the amplitude-normalized baseband signal can be cast as *s_b_*(*t*) = *I*(*t*) + *jQ*(*t*) = $\overset{K}{\underset{k=1}{\sum }}\exp\left(-j4\pi R_{k}\left(t\right)/\lambda \right)$, where the time-varying distances between the radar sensor and each of the *K* scatterers are $R_{k}\left(t\right)$ and *K* is the number of scatterers. Taking into account that the blade’s surface backscatters significant radar signals in the perpendicular direction, the distance between each scatterer and the radar makes the total received signals be coherently added. Therefore, its micro-Doppler signatures will have the form of flashes in the time-Doppler maps [28].

However, unique signatures with sinusoidal forms, called halos, are also observed in the spectrograms for rotating structures with translational velocity equals to zero (v = 0) [28]. The blade tips should be considered as point-scatterer targets. Considering that *R*_0_ is the nominal distance between the radar and the rotation center, *r* is the blade radius, and Ω is the angular velocity, the distance between the blade tips can be modeled as *R_tip_* = *R*_0_ + *rsin*(Ω*t*). For simplicity, the initial rotation angle on the rotation plane was suppressed. By applying the first derivative to the phase history of the complex baseband signal *s_tip_*(*t*) = $\exp\left(-j4\pi R_{tip}\left(t\right)/\lambda \right)$, the time-varying Doppler frequency of the blade tip is expressed as *f_D_*(*t*) = *−2*${R^{\prime }}_{tip}\left(t\right)$/*λ* = *−2r*Ω*cos*(Ω*t*)/λ, which demonstrates that the micro-Doppler signatures of the blade tips are theoretically sinusoidal. In fact, they appear as quasi-sinusoidal signatures in the time-Doppler maps due to the short-range detection [28].

**Figure 2:**
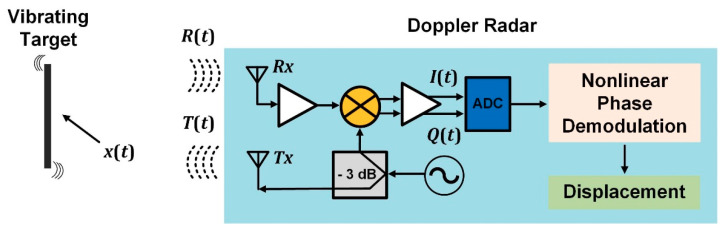
Block diagram for the SHM based on low-cost Doppler radars through remote vibration monitoring.

On the other hand, if the target presents a periodical linear motion, its displacement also provokes the phase-modulation of the previously transmitted radar signal. Figure 2 illustrates the block diagram for the remote vibration monitoring based on a Doppler radar system. Again, the backscattered reflected signal *R*(*t*) is mixed with the same transmitted signal *T*(*t*) by a quadrature-mixer to generate in-phase and quadrature-phase baseband responses. After the correct condition of the DC offsets, amplitude normalization, and circle fitting for the recorded *I*/*Q* baseband signals, the detected displacement can be estimated by nonlinear phase-demodulation algorithms, which combine both the in-phase and quadrature-phase signals to retrieve the changes in the phase angles of consecutive sampling points. Assume that the structure vibrates with a time-varying displacement *x*(*t*) = *msin*(*ω*_0_*t*), where *m* is the amplitude and the *ω*_0_ is the angular dominant frequency of the periodical motion. The two baseband responses can be written as *I*(*t*) *= sin*(4π*x*(*t*)/λ + *θ*) and *Q*(*t*) = *cos*(4π*x*(*t*)/λ + *θ*), where *θ* is the sum of the phase shift due to the nominal distance between the radar and the target, as well as the residual phase noise. By using arctangent demodulation, the vibration motion *x*(*t*) can be recovered as *x*(*t*) *=* (*λ/4π*) × {*arctan*[*I*(*t*)/*Q*(*t*)] − *θ*} = (*λ/4π*) × {*arctan*[*sin*(4π*x*(*t*)/λ + *θ*)/*cos*(4π*x*(*t*)/λ + *θ*)] − *θ*}, where *λ* is the wavelength of the transmitted RF signal [56]. Since the Doppler radars take advantage of the range correlation effect, the residual phase noise is negligible [57]. *θ* is only dependent on the constant nominal distance between the target and the radar. Therefore, it can be removed by subtracting the mean value.

Other transceiver architectures such as digital-IF receiver or double-sideband radars can also be utilized. Interested readers are encouraged to refer to [58,59,60] for practical discussions on different Doppler radar architectures.

## 3. Recent Advancements for SHM Based on Vibration Analysis

Portable Doppler/interferometry radar sensors are attractive for their robustness against ambient light and noncontact operation. Benefiting from the ability to provide superior accuracy in low-frequency displacement measurements, microwave Doppler radars have emerged as a potential solution for monitoring the health of civil infrastructures.

Board-level radars face limitations due to the lower transmitted power and the use of antennas with lower directivity. They have shorter detection range when compared with GBI radars. In recent years, researchers employed board-level Doppler radars either on the active backscattering mode or on the passive backscattering mode. In the active backscattering mode, active transponders placed at reference points are utilized to increase the power level of the radar’s received signal and then boost the signal-to-noise ratio (SNR) of the baseband signals. Although the installation of active transponders on the radar illumination scene might be complex, the maximum detection range can be increased, and it can be an alternative solution for the SHM of bridges with higher clearance. On the other hand, radars operating in the passive backscattering mode do not take advantage of active transponders to improve the SNR of the received signal. Digital signal processing strategies are utilized to essentially denoise the baseband signals or to calibrate the demodulated displacement. Disadvantages such as shorter detection range and less robustness against coupling clutter noises are apparent when the radar operates in the passive backscattering mode, since the received RF power is not boosted by corresponding active transponders. However, without the requirement of installing the transponders, the setup becomes considerably simpler for practical deployment.

### 3.1. Doppler Radar on Active Backscattering Mode

Researchers have studied the feasibility of using a board-level 2.4-GHz DC coupled Doppler radar to measure the dynamic and static displacement responses of bridges [61]. XBee radio modules were attached to the baseband board of the radar and to a laptop via a USB cable to enable wireless communication between the sensor and the computer, where the raw data was demodulated. Several laboratory experiments investigated the influence of the target distance, the motion amplitude, and the motion frequency on the accuracy of the displacement measurements. Although submillimeter accuracy was achieved for measurements in electronic lab environment, the authors observed the necessity of increasing the SNR of the baseband signals to boost the radar’s detection range.

**Figure 3:**
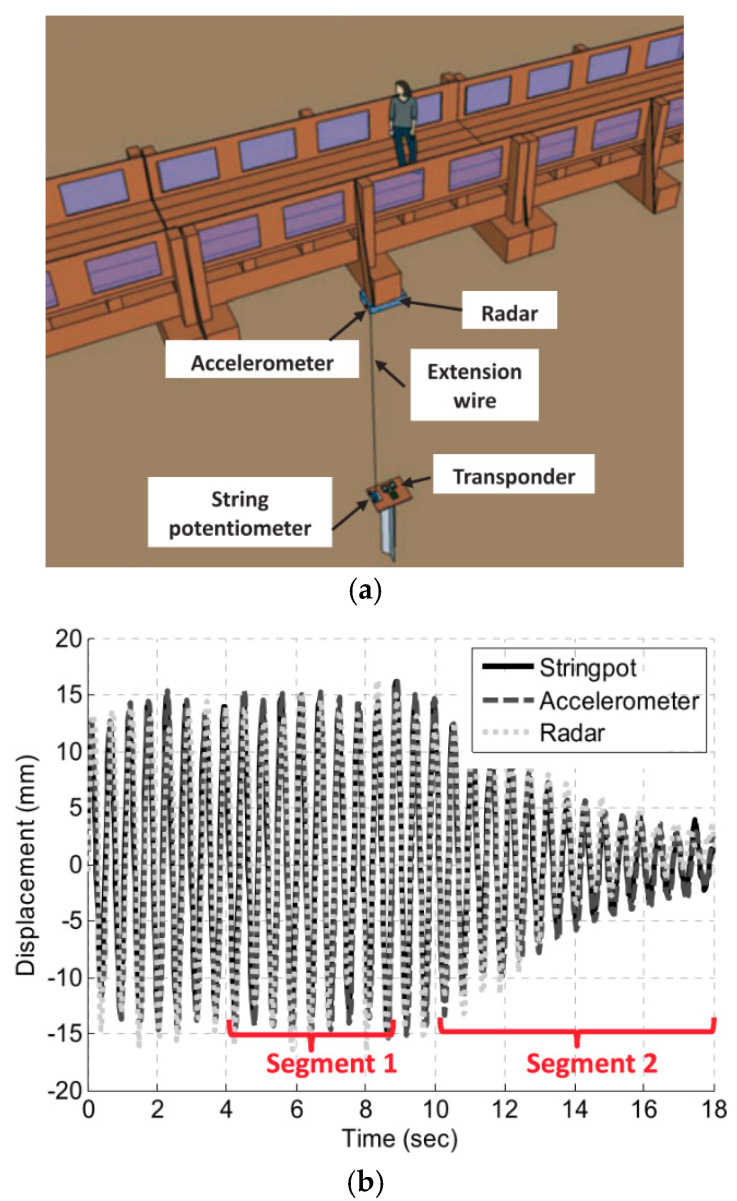
SHM of a bridge based on Doppler radar on active backscattering configuration: (**a**) experimental setup; (**b**) displacement measurements retrieved by all the sensors. Reprinted with permission from ref. [64]. Copyright 2016 John Wiley and Sons.

To solve the problem of the low SNR of baseband responses for longer monitoring range, the use of active transponders as reference points was considered to track the vibration measurements of a bridge [62,63]. In [64], full-scale tests were conducted on a 50-m long bridge at the O’Leno State Park, Florida, USA, as is revealed in Figure 3a. The employment of active backscattering configuration allows the increment of the received backscattered RF power or the change on the frequencies of the received signals. It also significantly reduced the influence of undesired surrounding clutter. Moreover, the use of high-directivity, bulky antennas became unnecessary, and the radars operating in the active backscattering mode can also be an alternative for the SHM of bridges with high clearance or be employed in scenarios with moving targets under the bridge such cars, boats or water flow. After various laboratory experiments demonstrated the improved performance of the active transponder configuration, a full-scale test was conducted to validate the proposed approach. The radar displacements were compared with the measurements obtained from a string potentiometer and an accelerometer. A transponder was placed on the ground 2.48 m below the radar. The vibration measurements were forced by a person jumping close to the quarter span with a frequency approximately equal to the first natural frequency of the bridge. Although the radar operation on the passive backscattering mode was not able to retrieve accurate measurement responses, the active backscattering mode for the SHM based on Doppler radar was successfully demonstrated. However, as the motion amplitude of the bridge decreased to less than 2 mm, the accuracy on the radar measurements deteriorated, as shown in Figure 3b (segment 2).

In contrast to the active transponder mode, which might be costly and is more power demanding than the passive backscattering mode, recent advancements on radar technologies leveraging nonlinear tags show alternative solutions for clutter rejection and target discrimination [65,66,67,68]. Although further investigations are required to demonstrate the feasibility of displacement reconstruction through radar detection aided by nonlinear tags, promising results on range tracking and physiological motion monitoring have paved the way for future SHM applications [65]. Recent works on passive nonlinear tags, which inherit the advantages of passive operation, low cost, and simple hardware, also demonstrated clutter rejection capabilities [66,67,68].

### 3.2. Doppler Radar Network on Active Backscattering Mode

**Figure 4:**
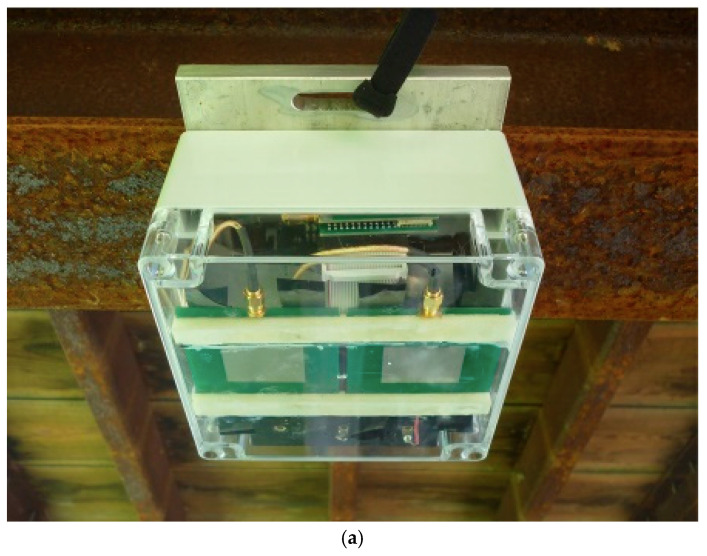

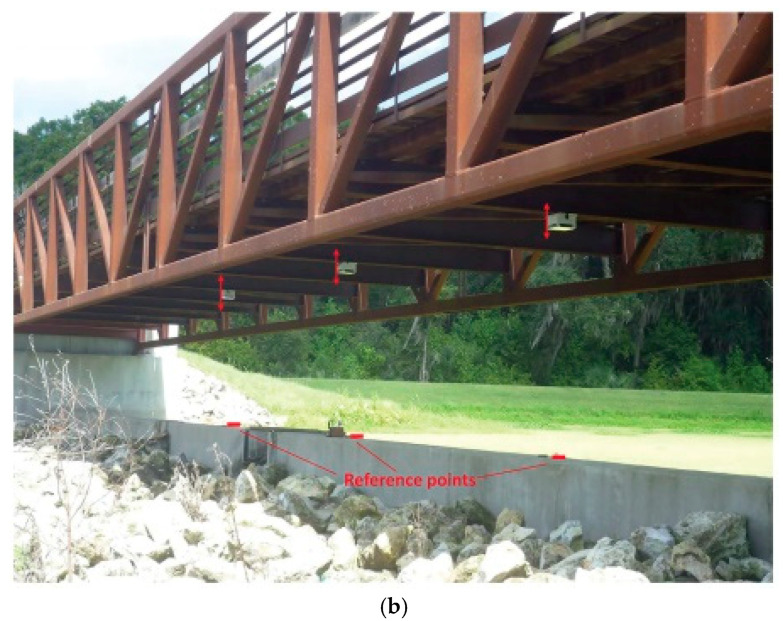
Experimental setup for the SHM of a bridge done by a Doppler radar sensor network: (**a**) Doppler radar mounted on plastic enclosure; (**b**) Doppler radar network deployed. Reprinted with permission from ref. [69]. Copyright 2018 American Society of Civil Engineers.

The compactness of board-level portable radars makes it also appealing to establish radar sensor networks to capture structural deflections at multiple locations. The simultaneous measurement of displacements at different parts of a structure are of paramount importance when structural modal analysis is needed. Researchers have proposed a wireless smart Doppler radar network to measure the dynamic and near static displacement of a 31.6 m long pedestrian steel girder bridge with a wood deck at Sweet Park in Gainesville, Florida, USA, as shown in Figure 4 [69]. The proposed smart Doppler radar sensor network operated in the active transponder mode. To address time-synchronization errors that may occur when a sensor network is deployed, and to account for the lack of onboard data processing capability of stand-alone Doppler radars, the authors had to employ a low-power wireless smart sensor platform, which was attached to each radar sensor node. Again, XBee radios were used as the wireless communication device for each radar and a central base station, which controlled the sensor network operation by sending commands to each sensor node. No communication was allowed among the radar sensor nodes to avoid interferences. Essentially, each node was intended to conduct the remote sensing during a predetermined duty cycle after receiving the command from the base station to preserve power. Then, the raw baseband signals were compressed and sent to the base station, where the analog radar signals were then automatically converted to displacement responses.

To deploy a smart wireless sensor network, it is also necessary to evaluate the power consumption of each node, since they rely on batteries. It was observed that the average current drawn for 1-h duty cycle was 52.9 mA for each radar node during 60 s of remote detection. In addition, the robustness of network operations depends on reliable wireless communication between the nodes and the base station. Since the XBee radios were utilized by each node of the network, the wireless transmission range for the XBee transceiver was investigated. The authors carried out transmission-range experiments with two smart Doppler radar enclosures mounted on a tripod at 1 m above the ground. The success rate of the transmission was defined as the number of sent packets that were received for a line-of-sight (LOS) link between two sensor nodes. The distance between the sensors continuously increased from 0 to 100 m in intervals of 10 m. After the mark of 100 m, the tests were done at intervals of 5 m. The transmission success rate of the XBee until 120 m was 100%. The success rate was higher than 90% for distances between 120 m and 150 m, and it eventually dropped quickly for distances greater than 150 m.

**Figure 5:**
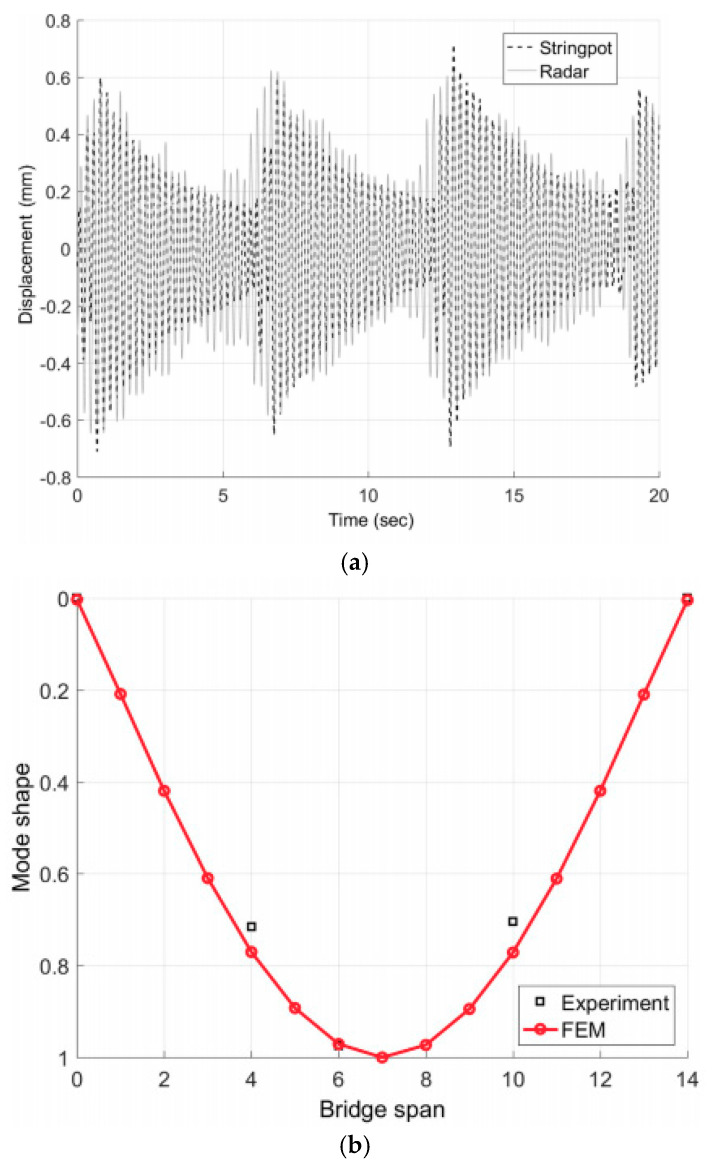
Experimental results for the Doppler radar sensor network operating in the active mode: (**a**) displacement measured at the midspan; (**b**) shape of the first mode. Reprinted with permission from ref. [69]. Copyright 2018 American Society of Civil Engineers.

To validate the proposed smart wireless network, three waterproof enclosures were assembled and then attached to the bottom of the bridge with the antennas directed towards the ground, which was approximated 1.7 m below the radars. One radar was installed at the midspan and the other two were placed close to the quarter-span. Three corresponding active transponders were installed directly below the radars on the ground. The base station was placed at approximately 15 m away from the closest radar. To verify the radar measurements at the midspan, a corresponding string potentiometer was set up. Initially, dynamically forced vibration was provoked at the midspan with an excitation frequency of 1.5 Hz. The natural frequency of the first vibration mode for the bridge is 4.7 Hz. Figure 5a exhibits the displacement measurement recorded at the midspan for the dynamic experiments and the corresponding ground truth (string potentiometer responses). The measurement errors were less than 0.1 mm for most of the experiments. However, for very small displacements (maximum amplitude <0.2 mm), the radar was not capable of providing accurate deflections. Furthermore, the first mode shape of the bridge was evaluated using the measurements from three deployed radars. A finite-element model (FEM) of the bridge provided the reference, and the three-point mode shape estimated by the radar responses were found to be in good agreement with simulation-based results as seen in Figure 5b. A near static deflection was excited by a 10-mph moving truck. By analyzing the deflections at the midspan and at the quarter-span after, the travel time between those two points was estimated as 1.01 s. Since the distance between them was 4.52 m, the approximate velocity of 9.99 mph was calculated for the moving vehicle.

Although wireless sensor networks contribute to minimize the implementation costs of SHM systems, significant time-synchronization errors may occur, which reduce the effectiveness of the use of multiple sensors for the simultaneous measurement recording on different locations. Furthermore, there was no consideration on the influence of environmental vibrations on the previously studied works. The motion analysis was always based on forced vibrations. Even with the presence of active transponders, which makes the scenario more complex and power demanding, the detection range of the proposed radar configuration (active backscattering mode) was limited to up to 3 m to provide reasonably accurate displacements.

### 3.3. Doppler Radar on Passive Backscattering Mode

**Figure 6:**
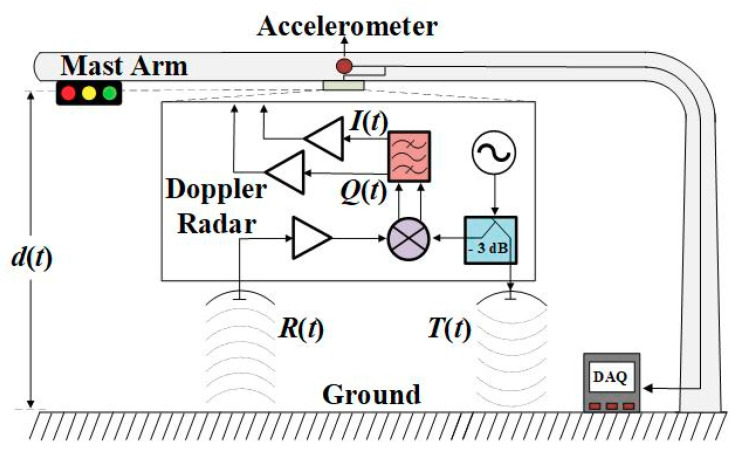
Block diagram for field structural health monitoring based on Doppler radar. Reprinted with permission from ref. [70]. Copyright 2020 IEEE.

Radars on passive backscattering mode can be easily set up and put in operation. Like radars on active backscattering mode, they can also be programmed for continuous monitoring or for the remote sensing from time to time to keep the power consumption low. To overcome the practical limitations of deploying active transponders, a feasibility study on the SHM of traffic light structure based on Doppler radar operating in passive mode was proposed [70]. The block diagram for the field SHM of a traffic light structure is revealed in Figure 6. For the first time, it was shown the presence of sudden jumps introduced in the oscillatory displacement obtained from the 5.8-GHz Doppler radar phase history. The major cause of this issue was the insufficient SNR of the received signal at times, caused by multipath effects and the long distance between the mast and the ground. Since the radar-based displacement is estimated by calculating the phase differences between successive sampling points, in moments of high noise levels, an abnormal point in the constellation graph may greatly deviate from the unit circle during the application of the conventional arctangent demodulation algorithm [56]. To address this issue, the non-adaptive joint signal processing algorithm (JSPA), which relies on the application of a median filter followed by a revise circle fitting, was proposed.

**Figure 7:**
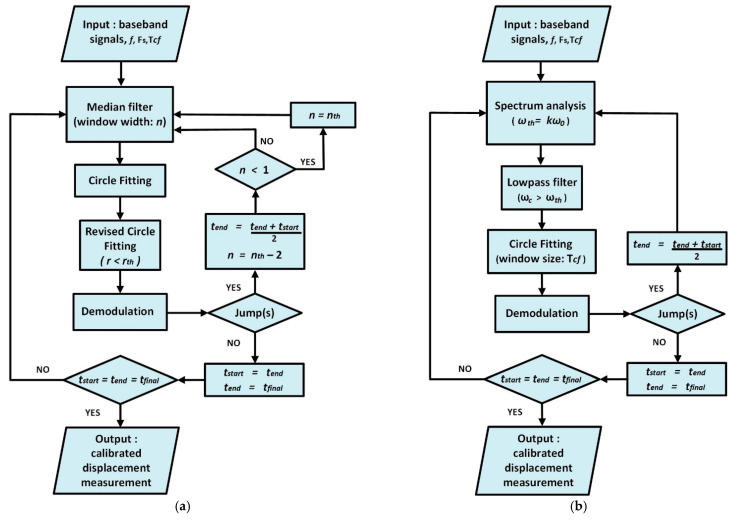
Flowchart for the propose adaptive displacement calibration strategies: (**a**) adaptive joint signal processing algorithm; (**b**) adaptive lowpass filtering algorithm. Reprinted with permission from ref. [71]. Copyright 2020 IEEE.

Since the manual adjustment of the parameters of the non-adaptive JSPA would impede its real-time implementation, two novel strategies were suggested to eliminate jumps in phase-demodulated Doppler radar data: the adaptive JSPA (AJSPA), which is the automated version of the JSPA, and the adaptive lowpass filtering algorithm (ALFA) [71]. The flowcharts of the proposed adaptive displacement techniques are shown in Figure 7. The AJSPA and the ALFA only requires as input a segment of the baseband signals, the sampling frequency, the radar operating frequency, and the window size of the circle fitting subroutine. The AJSPA first applies the median filter whose window size *n* depends on the used sampling rate. It contributes to reduce excessive noise levels and has an advantage of being more robust against a single corrupted point than the mean filter. Since jumps might be omitted by the median filter, a revised circle fitting subroutine is considered after the conventional circle fitting processing to ensure that remaining deviations are accounted for. On the other hand, the ALFA relies on a lowpass filtering approach, which automatically changes its relevant parameters according to the examined segment of raw radar data. Since the number of sinusoidal components on the *I*/*Q* baseband signals is a function of the instantaneous motion amplitude, the cut-off frequency of the adaptive lowpass filter embedded in ALFA is chosen to be slightly higher than the frequency of the weakest significant harmonic component. As a consequence, only the necessary harmonic components are preserved for the following phase-demodulation subroutine and high frequency interferences are attenuated.

The effectiveness of both approaches was validated by computer-generated and experimental results. Again, the mast arm was manually excited by pulling a rope until its motion reached large amplitudes. Then, the vibration decayed freely. The radar was mounted near the midspan and the ground truth was simultaneously detected by a conventional tri-axial DC-response accelerometer of ±4 g in measurement range that was installed to the same axial position as the radar. The *I/Q* channels of the radar and the channels of the accelerometer were digitized by a National Instrument NI-9239 voltage input module on a CompactRIO platform, used as the data acquisition system (DAQ).

**Figure 8:**
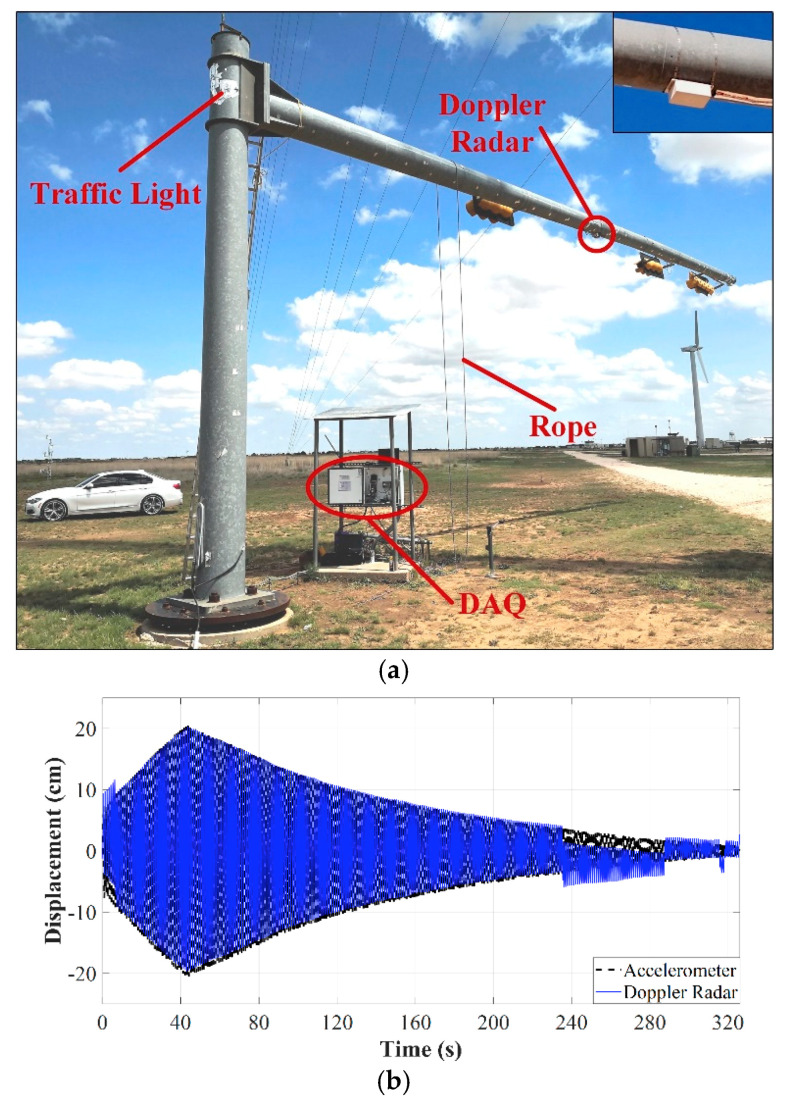
Experimental results for the field SHM of a traffic light structure: (**a**) experimental setup with the radar enclosure shown in the inset; (**b**) demodulated displacement using only arctangent demodulation. Reprinted with permission from ref. [71]. Copyright 2020 IEEE.

**Figure 9:**
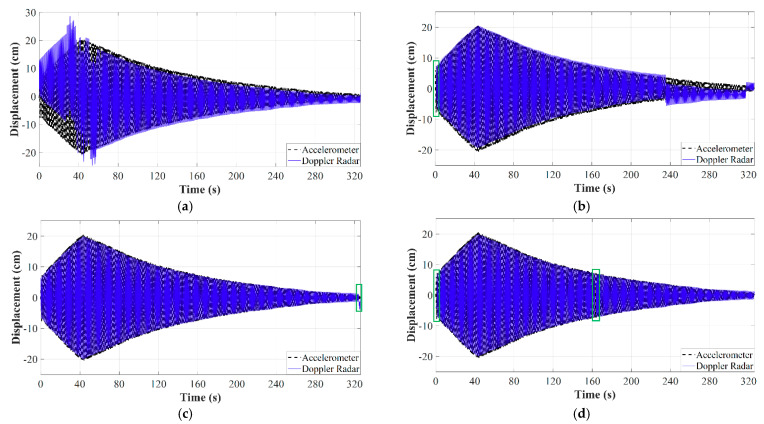
Experimental results: (**a**) demodulated displacement using the non-adaptive JSPA; (**b**) demodulated displacement using the non-adaptive ALFA; (**c**) demodulated displacement using the AJSPA; (**d**) demodulated displacement using the ALFA. Discontinuities are highlighted in (**b**–**d**). Reprinted with permission from ref. [71]. Copyright 2020 IEEE.

Figure 8 depicts the experimental setup and the displacement obtained after the use of the conventional arctangent demodulation algorithm on one of the recorded data sets. Jumps are clearly seen around instants 240 s and 320 s. The manual versions of the AJSPA and the ALFA were also implemented in MATLAB and applied to the recorded data set shown in Figure 8b. Figure 9a,b illustrate the displacement measurement obtained through the non-adaptive JSPA and the non-adaptive lowpass filtering technique, respectively. The accelerometer measurements are compared with the radar responses. Discontinuities and jumps are observed in both demodulated data, which demonstrates that the non-adaptive strategies are not robust for practical SHM applications. The reason the non-adaptive JSPA fails in providing accurate displacements is because it keeps the window size of the median filter constant, which causes severe attenuation on high-frequency components particularly when the amplitude motion is considerably large. The non-adaptive lowpass filtering strategy is ineffective because, as the motion amplitude continuously changes, the number of significant harmonics also changes, while the cutoff frequency of the lowpass filter is fixed. Thus, it does not account for amplitude motion variations. In contrast, Figure 9c,d reveal the radar displacements after applying the AJSPA and the ALFA, respectively. The sudden bounces are completely removed from the demodulated Doppler radar data since both approaches modify its key parameters as the amplitude motion changes. However, although the radar measurements were in good agreement with the ground truth, when the SNR deteriorates, the AJSPA produced distortions as seen in Figure 9c. Likewise, discontinuities caused by the process of cutting apart displacement with abrupt jumps are highlighted in Figure 9d. The RMS error for the AJSPA response was 0.445 cm and the RMS error between the ALFA measurement and the reference was 0.465 cm. The authors also analyzed the proposed algorithms regarding their accuracy and computational demand for other seven recorded data sets. Both calibration strategies provided sub centimeter measurement accuracy. During the computational complexity analysis, ALFA performances presented higher latencies than the AJSPA performances since the latter has an embedded spectral analysis subroutine. The proposed adaptive calibration strategies are capable to handle the issue of introduced sudden jumps and can be used for the data demodulation of long-term baseband signals. However, without the presence of active transponders or nonlinear tags, clutter and moving targets remained a challenge for accurate SHM based on Doppler radar operating in passive backscattering mode.

### 3.4. Doppler Radar Array on Passive Backscattering Mode

**Figure 10:**
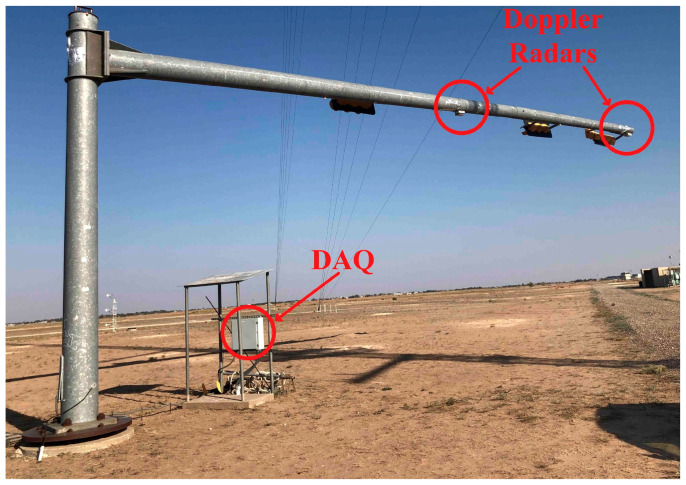
Experimental setup. The low-cost Doppler radar array and the data acquisition instrumentation are highlighted [72].

**Figure 11:**
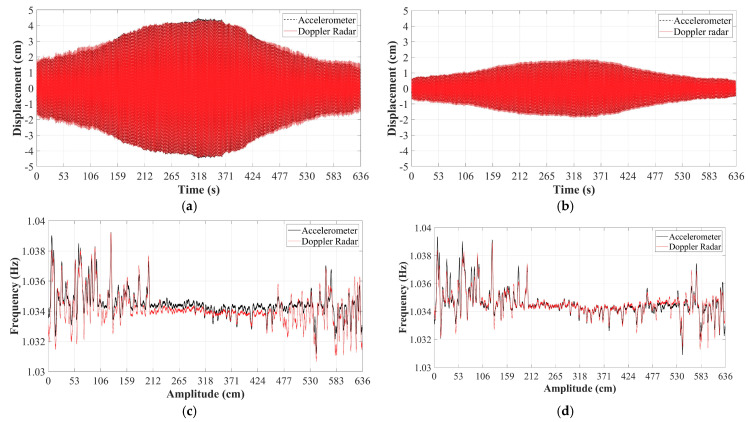

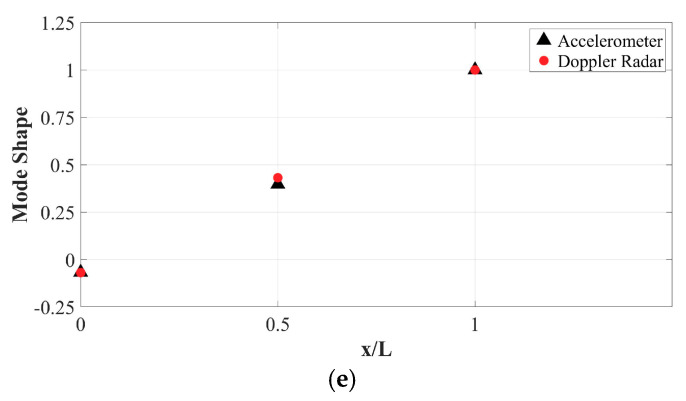
Experimental results: (**a**) demodulated displacement using the AJSPA retrieved at the tip of the mast; (**b**) demodulated displacement recovered at the midspan; (**c**) instantaneous dominant frequencies at the tip; (**d**) instantaneous dominant frequencies at the midspan; (**e**) first in-plane mode shape of the traffic light structure evaluated by the Doppler radar array and an accelerometer attached to the pole-arm connection [72].

As already mentioned, the structural modal analysis is obtained from the synchronous multipoint vibration measurements. In this paper, cabled radar networks, which do not rely on batteries as DC power sources, are referred to as radar sensor arrays. By studying the changes on the vibration mode shape, another important parameter for the structural health diagnosis can be evaluated. Recently, researchers proposed a low-cost Doppler radar array operating in the passive backscattering mode for the SHM of a traffic light as illustrated in Figure 10 [72]. In the full-scale tests, forced vibrations demonstrated that the displacements at the tip and at the midspan could be simultaneously recovered with sub centimeter accuracy. The references were displacements estimated by twice numerical integration of the corresponding accelerometer measurements. A high-pass filter with a cut-off frequency of 0.5 Hz was applied to the processed data after each integration step. In contrast to most of the existing SHM works based on Doppler radars, structural response to environmental loads was also considered. The arrays were prepared to uninterruptedly monitor the traffic light for several days. Only the segments of raw data that produced displacements larger than ~1 cm at the tip and larger than ~0.5 cm at the midspan were successfully phase-demodulated. The amplitude motion significantly affects the SNR of the received RF signals [73]. Since jumps were seen at the arctangent demodulated data retrieved from the tip of the mast, the previously mentioned AJSPA was necessary to calibrate the radar measurements. Figure 11a,b show the wind-induced vibration measurements obtained at the tip and at the midspan, respectively. The RMS error for the displacement recovered at the tip was calculated as 0.282 cm. The error for the displacement measured at the midspan was 0.117 cm, which was smaller due to the introduced distortions by the filtering approaches embedded in the AJSPA. Fourier-based analysis and structural modal evaluations obtained through proper orthogonal decomposition algorithms are not able to capture the non-stationary fluctuations caused by environmental vibrations because these methods only deliver the average spectral decomposition of the signal [74,75]. On the other hand, multi-resolution strategies that rely on complex Morlet wavelet transforms can overcome these limitations [76,77,78,79]. Therefore, the authors estimated the evolution of the dominant vibration frequencies by identifying the frequencies associated with the maximum magnitude of the wavelet scalograms of the measurements obtained from Doppler radar and accelerometer displacement data at every instant. Figure 11c,d exhibit the plots for the instantaneous natural frequencies retrieved at the tip and the midspan, respectively. The variance on the dominant vibration frequencies is relatively higher for the smaller amplitude motion, and it turns lower when the amplitude of the mast movement increases. The RMS errors for the dominant frequencies estimated by the Doppler radar array were 0.0006 Hz for the measurements retrieved at the tip of the mast and 0.0003 Hz for the measurements recovered at the midspan. Again, the use of the AJSPA to calibrate the demodulated displacement at the tip affected the accuracy for the calculated dominant frequencies.

By including the vibration measurements retrieved by an accelerometer placed at the pole-arm connection of the traffic light, the first in-plane structural mode was also calculated. A time-domain POD method was applied to the modal responses simultaneously recorded at three different locations of the mast (tip, midspan, mast-pole connection). In summary, the high sensitivity of a Doppler radar array to shifts on the instantaneous natural frequencies was demonstrated. In addition, the estimation of the shape of the first in-plane mode for a traffic light structure was obtained through measurements conducted by radars operating in the passive backscattering mode. Although the use of signal processing techniques handled the issue of the low SNR at times without the need of active transponders, the proposed work only reported sub centimeter accuracy results and the radar measurement errors for very small vibrations (less than 0.5 cm) were notably significant. Furthermore, a cabled apparatus was deployed, which might be a costly and non-practical solution for certain SHM applications.

## 4. Recent Advancements for SHM Based on the Analysis of Time-Doppler Signatures

Among all the available renewable energy sources, wind energy is the one with the widest adoption in the United States and many other countries in the world [80]. With the increasing production of wind energy from large turbines and larger wind farms, real-time monitoring of operating wind turbines is of critical importance to minimize the maintenance cost, reduce potential costs due to early damage, and avoid human and animal life-threating events.

**Figure 12:**
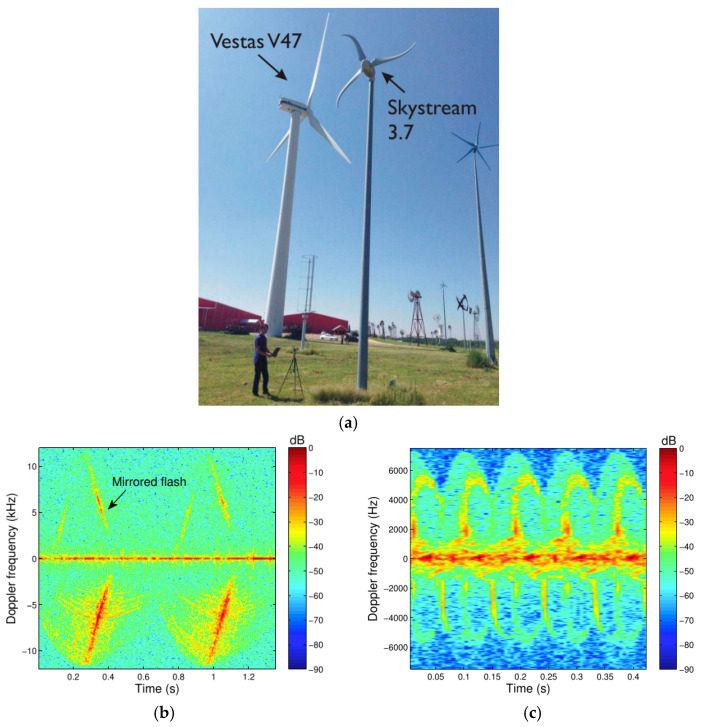
SHM of industrial wind turbines: (**a**) experimental scenario; (**b**) spectrogram for the Vestas V47 illuminated by a 24-GHz Doppler radar; (**c**) spectrogram for the Skystream 3.7 illuminated by a 24-GHz Doppler radar sensor. Reprinted with permission from ref. [28]. Copyright 2016 IEEE.

Due to the capability of also providing time-Doppler signatures, radars have been investigated for SHM of wind turbines [25,26,27,28,81,82,83,84,85,86,87,88,89,90,91,92,93]. The long-term, long-range, and compactness of low-cost radar sensors make them strong candidates to the remote detection of the blade’s motion. The theoretical study of Doppler radar signatures acquired by short-range Doppler radars and a parabolic model for blade curvature were proposed in [28]. Through mathematical simulations, the different aspects of the time-Doppler responses with respect to contrasting blade forms and radar illumination angles (0° and 90°) were analyzed. The authors concluded that the curved flashes obtained after the detection of moving curved blades are the result of the coherent sum of signal contributions associated with blade scatterers, i.e., the flashes exist due to the constructive interference of equal-phase scatterers not located at the blade tip, and the halos are related to the movement of the blade tip. To experimentally validate the theoretical results, radar measurements were carried out for a 50-m-height (660-kW Vestas V47) and a 12-m-height (1.9-kW Skystream 3.7) wind turbine in the American Wind Power Center, Lubbock, TX, USA, as illustrated in Figure 12a. Two Doppler radars operating in the C-band (transmitting frequency at 5.8-GHz) and the K-band (transmitting frequency at 24-GHz) frequencies were deployed. By observing the positive and negative Doppler flashes, one can estimate the blade rotation period, and then its angular velocity, which is not only a function of the wind speed but is also roughly correlated with the amount of power that will be converted into electricity. The main difference between the two Doppler radars employed in this study are the transmitting RF frequency and the antenna beamwidth. For the 24-GHz radar, the detected turbine blades were not completely contained within the antenna beam. However, the higher operation frequency enabled the construction of higher resolution spectrograms. To better benefit from the radial speed detection of Doppler radars and mitigate spurious curvature effects, illumination angles near to zero are preferred. In addition, the proposed parabolic model for curved blades was verified by simulations and confirmed by the experiments conduct with the Skystream 3.7 wind turbine. Figure 12b shows the spectrogram for the Vestas V47 illuminated by a 24-GHz Doppler radar. The positive and negative-Doppler flashes, which are associated with the different contributions of the blade segments when their instantaneous position is perpendicular to the transmitting radar signal, are confirmed. It should be noted that the presence of mirrored flashes is attributed to the *I*/*Q* mismatches of the in-house mixer used in the 24-GHz radar prototype. Figure 12c exhibits the spectrogram for the Skystream 3.7 when illuminated by a 24-GHz Doppler radar sensor. The hook shape of the flashes validates the proposed parabolic model for curved blades. An estimation of the blade curvature can be provided through the analysis of the radar signatures. Finally, the authors did not report any approaches or methods for the eventual SHM of the blades. Only theoretical findings were demonstrated by computational results and realistic experiments.

**Figure 13:**
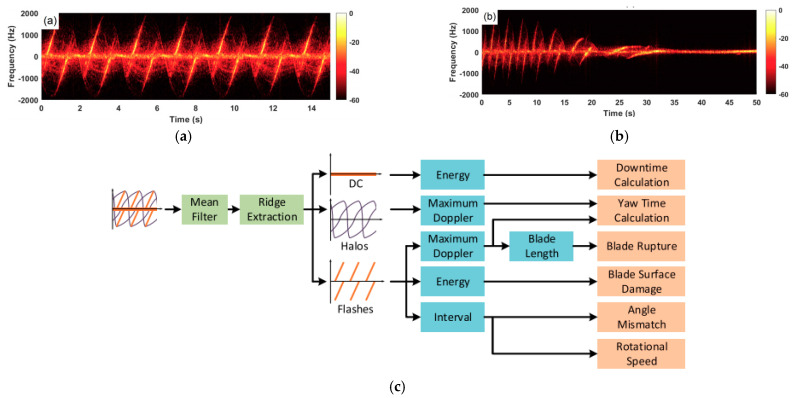
SHM of a onshore wind turbine: (**a**) spectrogram when the turbine is fully operational; (**b**) spectrogram when the blade rotation is gradually stopping; (**c**) flowchart of the proposed strategy for the SHM of a wind turbine. Reprinted with permission from ref. [94]. Copyright 2021 IEEE.

A novel strategy for the structural condition monitoring of a horizontal axis wind turbine was proposed in [94]. The solution relied on the extraction of three key parameters (rotational speed, downtime, and duration of yaw) from the time-Doppler map associated with the turbine blades’ movement. Figure 13a,b reveal the spectrograms when the turbine is fully operational and when it is gradually stopping. The rotation speed is related to the amount of generated power as already mentioned. The monitoring of downtime, which is the time of reduced operation or inactivity, is also associated with energy generation. The yaw monitoring provides information about the blades’ condition. The structural health diagnosis of the wind turbine is assessed by analyzing the spectral energy, the maximum detected Doppler frequency, the time interval between flashes after the separation of the DC level, the halos, and the flash signatures from the spectrogram. The proposed algorithm calculates the downtime from the overall estimated spectral energy at the DC level. If the energy at the zero-Doppler suppresses a given threshold for more than 5 s, the counter for the downtime is triggered. The yaw time evaluation is based on the maximum Doppler speed. Again, if the blades are not perpendicularly oriented towards the radar due to changes on the direction of the wind, the maximum detected frequency will vary, and the duration of this event can be measured. The blade rupture can easily be identified by observing the maximum Doppler frequency for each blade. Blade surface damage can also be detected by the analysis of the processed time-Doppler signatures. Since the inner constituents of the blade have different backscattering properties, the reflected echoes (flashes and halos) of blades with surface damages would be less powerful. Angle mismatches are observed when the inclined angle between successive blades changes, which indicates that the blade is deviating from its original position. The interval time between the corresponding flashes can also be used to estimate the rotational speed. Figure 13c illustrates the proposed procedure for extracting SHM features from the corresponding time-frequency plots.

**Figure 14:**
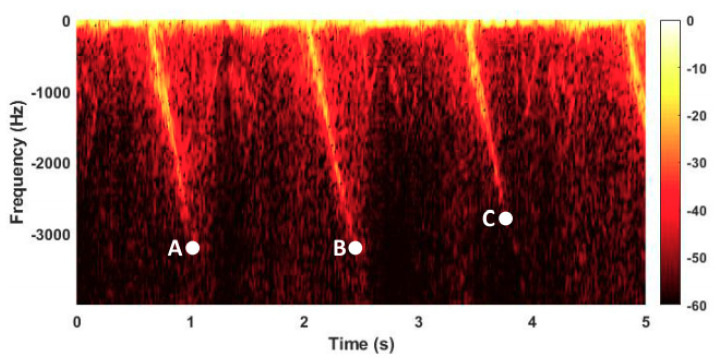
Spectrogram for a faulty three-blade wind turbine obtained from 5.8-GHz Doppler radar measurements. Reprinted with the permission from ref. [94]. Copyright 2021 IEEE.

Simulations and experimental results obtained through the radar monitoring of real turbines from a wind farm were used to demonstrate the proposed strategy. The blade length for each turbine is 55.5 m. A 5.8-GHz Doppler radar was chosen to illuminate the targets. Five wind turbines were monitored for the rotational speed, which was compared with a reference extracted from simultaneously recorded videos. The measurement error after two trials for each turbine remained below 0.4 rotations per minute. They also calculated the downtime for a gradually stopping turbine. The radar-based measurement was 21.31 s and the reference was given as 19.4 s. Finally, a faulty diagnosis was performed. After the calculation of the maximum Doppler frequencies for the flashes associated with the monitored blades, it was observed that one of them had a rupture since its speed greatly deviated from the other two. Figure 14 shows an extract of the spectrogram for the three-blade faulty wind turbine. The Doppler frequencies on points A and B are, respectively, −3230 Hz and −3208 Hz. However, the frequency on point C is −2864 Hz, which flagged a damaged blade. Since the features’ extraction depends on the acquired spectrograms, the alignment of the radar with the rotation plane of the blades plays a critical role. For example, if the radar’s field of view is not identical for receding and approaching blades, the algorithm will yield false positive results. The summary list of the research works reviewed in this paper is given in Table 1.

## 5. Conclusions

Powered by the advancements of semiconductor technologies, Doppler radar can be miniaturized, which led to vast practical implementations in the civilian world. The recent technical advancements on digital signal processing pushed the technology further into smart integration with embedded systems. In addition, the potential costs of Doppler radar sensors after CMOS integration and mass production would possibly make them even more appealing than other noncontact approaches for SHM such as camera systems and laser vibrometers. Beamforming technology can also be exploited to address the current issue of measuring only the displacement on the radial direction. Furthermore, by leveraging the compactness of low-cost Doppler radars, radar sensor networks can be deployed. Although the adoption of low-cost, compact radars still lags behind other technologies, SHM researchers and engineers can be optimistic about the promising possibilities for low-cost radars with the rapid dissemination of chipset-based sensors and migration to mm-wave bands.

## Figures and Tables

**Table 1:** Summary list of the research works reviewed in this paper.

Radar System/Arrangement	Mode	Publication Year	Testbed	Type of Vibration	Reported Range	Meas. Accuracy
Multi-monostatic GBI radar [55]	Passive backscattering	2021	Bridge monitoring	Forced	23 m/33 m	<1 mm
2.4-GHz Doppler radar [61]	Passive backscattering	2015	Aluminum beam	Forced	1.25 m	<1 mm
2.4-GHz Doppler radar [64]	Active backscattering	2016	Pedestrian bridge	Forced	2.48 m	<1 mm
2.4-GHz Doppler radars arranged as a wireless network [69]	Active backscattering	2019	Pedestrian/car bridge	Forced	4.52 m	<1 mm
5.8-GHz Doppler radar [70]	Passive backscattering (non-adaptive displ. calibration algorithm)	2020	Traffic light structure	Forced	6 m	<1 cm
5.8-GHz Doppler radar [71]	Passive backscattering (adaptive displ. calibration algorithms)	2020	Traffic light structure	Forced	6 m	<1 cm
5.8-GHz Doppler radars arranged as an array [72]	Passive backscattering	2021	Traffic light structure	Forced & ambient vibration	6 m	<1 cm
5.8-GHz/24-GHz Doppler radar [28]	Passive backscattering	2016	Wind turbine	Forced	Rotor diameter: 47 m/3.7 m	-
5.8-GHz Doppler radar [94]	Passive backscattering	2021	Wind turbine	Forced	Rotor diameter: 111 m	-
